# Study on Performance Simulation of Vascular-like Flow Channel Model Based on TPMS Structure

**DOI:** 10.3390/biomimetics8010069

**Published:** 2023-02-06

**Authors:** Jianping Shi, Fuyin Wei, Bilal Chouraki, Xianglong Sun, Jiayu Wei, Liya Zhu

**Affiliations:** 1 School of Electrical and Automation Engineering, Nanjing Normal University, Nanjing 210046, China; 2 Division of Sports Medicine and Adult Reconstructive Surgery, Department of Orthopedic Surgery, Nanjing Drum Tower Hospital, Nanjing 210008, China

**Keywords:** vascular flow channels, TPMS structures, fluid-solid coupling, 3D bioprinting

## Abstract

In medical validation experiments, such as drug testing and clinical trials, 3D bioprinted biomimetic tissues, especially those containing blood vessels, can be used to replace animal models. The difficulty in the viability of printed biomimetic tissues, in general, lies in the provision of adequate oxygen and nutrients to the internal regions. This is to ensure normal cellular metabolic activity. The construction of a flow channel network in the tissue is an effective way to address this challenge by both allowing nutrients to diffuse and providing sufficient nutrients for internal cell growth and by removing metabolic waste in a timely manner. In this paper, a three-dimensional TPMS vascular flow channel network model was developed and simulated to analyse the effect of perfusion pressure on blood flow rate and vascular-like flow channel wall pressure when the perfusion pressure varies. Based on the simulation results, the in vitro perfusion culture parameters were optimised to improve the structure of the porous structure model of the vascular-like flow channel, avoiding perfusion failure due to unreasonable perfusion pressure settings or necrosis of cells without sufficient nutrients due to the lack of fluid passing through some of the channels, and the research work promotes the development of tissue engineering in vitro culture.

## 1. Introduction

Successful biological tissue engineering fabrication in current studies is limited to thin tissues such as the cornea, skin, and bladder. Due to limitations such as oxygen diffusion, it is difficult to maintain the survival of large tissues thicker than 200 µm, especially for tissue-like organs with high volumetric oxygen consumption rates (e.g., heart-like, pancreas, and liver tissues), and the progress of large-scale tissue engineering is seriously hampered by the lack of necessary vascular network systems in the fabricated such tissues. Therefore, the fabrication of functional bionic tissues needs to consider the construction of multi-scale vascular networks, and even some tissues need to incorporate lymphatic and neural networks. Most of the currently bioengineered bionic tissues lack these transport networks. The 3D printing technology is capable of directly manufacturing complex multi-material structures with controllable shape structures and controllable material components and has become the primary choice for tissue engineering manufacturing.

Three-dimensional bioprinting technology involves combining various disciplines based on additive manufacturing techniques, allowing the printing of tissues or organs with mechanical properties, antibacterial properties, biological stability, and transplantation capabilities to meet patients’ needs, with the ultimate goal of printing organs such as specific organs or artificial blood vessels. The fabrication of human-like tissue and organ structures using 3D bioprinting has been a hot topic of research in the field of biomedical engineering [1,2]. Zhu et al. constructed 3D microscale flow channel networks containing complex structures by using a light-curing approach [3]. Griffith et al. printed a liver containing blood vessels by using inkjet 3D printing technology [4]. Ma et al. printed liver lobular structures containing vascular networks using a light-curing approach [5]. Yang et al. proposed a light curing method that allows the fabrication of 3D vascularized channels of different shapes within a hydrogel matrix to maintain thick tissues in culture [6]. The difficulty in further culturing 3D bioprinted vascular flow tissues in vitro is to provide sufficient oxygen and nutrients to ensure proper cell growth [7]. In vitro culture of tissue engineering requires the simultaneous growth of an internal vascular flow pathway system to ensure the proper exchange of nutrients, air, and metabolic waste between the cells and the internal environment [8,9]. Diffusion is the method used in most tissue culture projects [10]. Perfusion culture provides fresh culture fluid for tissue engineering and allows diffusion, which is an effective means of promoting normal cell growth. Therefore, for 3D bioprinting of vascular flow paths, in vitro perfusion culture is the most effective method for bio-tissue engineering [11,12]. In recent years TPMS (Triply Periodic Minimal Surfaces) porous structures have been widely used in biological tissue engineering [13,14]. TPMS is a minimal surface that exhibits periodicity in three independent directions in three-dimensional space. The surface has the characteristics of zero mean curvature and can be extended indefinitely in three periodic directions. It can provide a concise description of many physical structures, such as silicates and soluble colloids. As a pore-forming unit, TPMS can realize the digital representation of a porous structure. There are also advantages to the porous structures found in capillaries, such as diffusion of nutrients and small pore size limitations [15]. Davar Ali investigated the effect of TPMS scaffold structures on cell adhesion during the diffusion of nutrient delivery in culture media [16]. Fluid flow dynamics transported basal nutrients to cells, and TPMS porous structures had greater fluid permeability than other structures, which facilitated cellular metabolic activity [17]. The permeable TPMS porous network structure facilitates the wetting of the culture medium and contributes to the diffusion of nutrients [18].

A major challenge in engineered tissue fabrication is the structure of an integrated complex hierarchical vascular network similar to that found in natural tissues spanning arteries and veins down to the smallest capillaries. The following two main types of research directions have been developed: the construction of microchannels to improve the diffusion of nutrients and oxygen and the formation of vascular growth factors in tissue structures to promote vascular development. The construction of microchannels is a necessary prerequisite for the smooth transport of nutrients and wastes required for the growth of various types of cells in tissues and organs, and it can enable the manufactured tissues and organs to be successfully cultured and shaped so how to establish a network flow channel organization that structurally and functionally resembles the native vascular system is a current research hotspot. In this study, we propose to simulate and analyze the internal flow paths in the tissue model.

In contrast, most of the simulation analyses of the perfusion pressure of the bionic tissue flow channel are simulations of simple 3D vascularised flow channels as in the literature [19] or the use of porous TPMS structures for bionic bone tissue engineering [20], however very few involve the use of TPMS porous structures in vascularised flow channels. The relevant studies mentioned above have less often involved the analysis of the perfusion pressure of the bionic tissue flow channels during the process of thick tissue fabrication, so the analysis of the perfusion pressure of the bionic tissue flow channels of TPMS porous structures needs to be performed during the process of thick tissue fabrication, to ensure high viability of the manufactured bionic tissues when cultured in vitro, the appropriate perfusion pressure is extremely important when using a pressure perfusion device to perfuse a bionic tissue flow channel model. If the perfusion pressure is too high, the fluid flow rate will be too fast, resulting in greater pressure on the model channel, greater deformation of the model channel, and even collapse of the tissue; if the perfusion pressure is low, the fluid flow rate will be inadequate, preventing cell metabolism from taking place and the cells from achieving the appropriate results. In addition, the amount of fluid flowing through the bionic tissue flow channel is also extremely important; if there is little to no fluid passing through a certain area of the bionic tissue, the necessary nutrients are not available, and the tissue cannot be cultured. Based on this, this paper designs a TPMS-based model of the bionic tissue flow channel and simulates the characteristics of the fluid in the model flow channel to obtain optimal parameters for the smooth cultivation and survival of the bionic thick tissue.

## 2. Materials and Methods

### 2.1. Vascular-like Flow Channel Modeling

There are many ways to generate TPMS coordinates, the approximate TPMSs periodic surface is generally defined as follows:

(1) $$\emptyset \left(r\right)=\sum_{k=1}^{K}A_{k}\cos\left[\frac{2\pi \left(h_{k}\cdot r\right)}{\lambda_{k}}+p_{k}\right]=C$$

where *γ* represents position vectors of Euclidean space, $A_{k}$ represents the amplitude factor, $h_{k}$ is the kth grid vector in the reciprocal space, $\lambda_{k}$ represents the periodic wavelength, $p_{k}$ represents the phase offset, and C is a constant.

In this article, the P-shaped surface is used as the construction unit for the construction of the bionic flow channel model, then its construction equation can be expressed as follows:

(2) $$f_{p}\left(x,t\right)=\cos\left(x\right)+\cos\left(y\right)+\cos\left(z\right)+0.5$$

**Figure 1:**
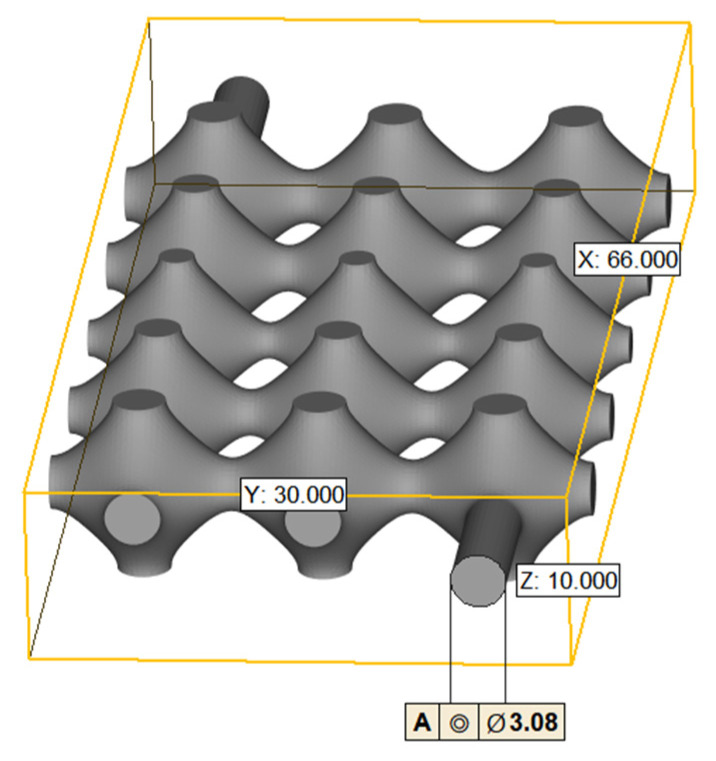
TPMS vascular flow path model.

We use nTopology (nTopology, New York, NY, USA), a parametric design software, to generate TPMS visualization .stl format files for P-shaped structures, the dimensions of the constructed TPMS flow channel model is shown in Figure 1 as follows: the diameter of the inlet and outlet is 3.08 mm, the overall length of the model is 66 mm, the width is 30 mm and the height is 10 mm. The volume is 4739.85 ${\mathrm{mm}}^{3}\;$and the area is 3662.961 ${\mathrm{mm}}^{2}$.

### 2.2. Simulation Analysis

**Figure 2:**
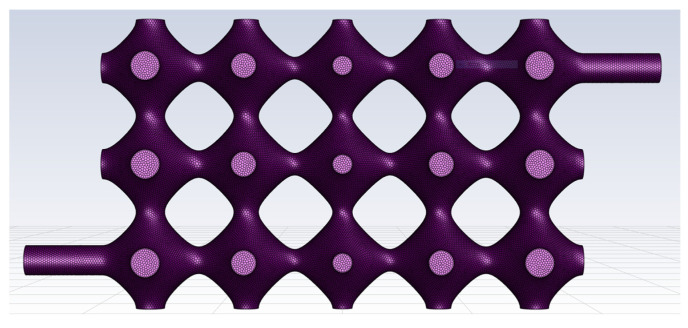
Class vascular flow path model grid.

The TPMS vascular flow channel model was first meshed using Fluent Meshing, the fluid meshing tool that comes with Fluent 2020 R2. The cell type used for the automatic meshing volume filling was poly, and the meshing of the vascular-like flow channel model is shown in Figure 2.

**Figure 3:**
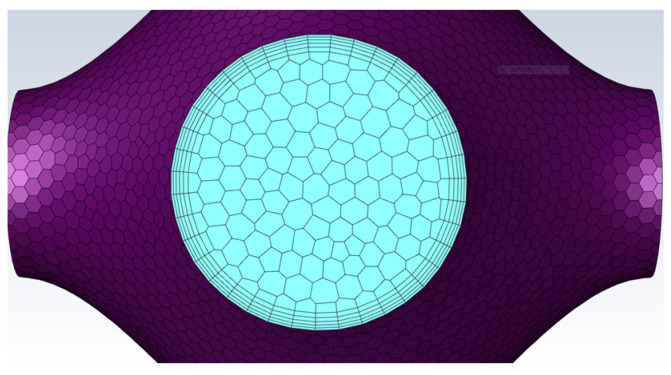
Boundary layer along the wall.

It is worth paying attention that the TPMS vascular flow channel model is a thin-walled structural unit, and the boundary layers along the wall affect the state of the fluid, so it is important to have a boundary layer mesh at the wall. In this paper, five boundary layers are set up at the wall surface, as shown in Figure 3.

The finite element simulation of the fluid meshing model is based on the analysis of both blood flow velocity and vessel wall pressure; therefore, a pressure-based solver is used. The pressure solver is based on the momentum equation and the continuity equation, which are used to derive the blood flow rate and vessel wall pressure. The momentum equation is shown in Equation (3).

(3) $$\frac{\partial }{\partial_{t}}\left(\rho u_{i}\right)+\frac{\partial }{\partial_{x_{j}}}\left(\rho u_{i}u_{j}\right)=-\frac{\partial_{p}}{\partial_{x_{i}}}+\frac{\partial_{\tau_{ij}}}{\partial_{x_{j}}}+\rho g_{i}+F_{i}$$

where: $\tau_{ij}$ is the stress tensor, p is the static compressive force, and $g_{i}$ and $F_{i}$ respectively i represent the gravitational volume force and the external volume force in the direction.

The continuity equation is given in Equation (4), where ρ is the density of the fluid (kg/$m^{3}$), and A is the effective cross-sectional area ($m^{2}$), and v is the average velocity over the effective section (m/s).

(4) ρAv=C

In the case of an incompressible fluid, the ρ is a constant the continuity equation is then Equation (5)

(5) $$A_{1}V_{1}=A_{2}V_{2}$$

For ease of analysis and accuracy of calculations, the temperature and viscosity of the blood are not taken into account, which means the blood is of constant characteristics. At different Reynolds numbers, the flow state of the fluid varies. The state of blood flow within the vascular-like flow channel is determined by calculating the Reynolds number. The Reynolds number within a vascular-like flow channel can be described by Equation (6).

(6) $$R_{e}=\frac{v_{b}*D*\rho }{\mu }$$

where $v_{b}$ is the velocity of the fluid; D is the diameter of the flow channel.ρ is the density of the fluid. μ is the dynamic viscosity of the fluid. $v_{b}\;$, *D*, ρ and μ can be chosen from 0.1 m/s, 3.08 mm, 1060 kg/$m^{3}$ and 0.005 Ns/$m^{2}$, refer to the blood physiological parameters. From Equation (6) it can be calculated that the Reynolds number is equal to 63.6, which is less than 2300. Therefore, the blood flow state in the vascular-like flow channel can be chosen as laminar flow. The governing equation for incompressible laminar Newtonian fluid is the Navier–Stokes equation as follows [21].

(7) ∇U=0

(8) $$\rho U\times \nabla U=\nabla (-p+\mu (\nabla U+(\nabla {U)}^{T}))$$

where p is the pressure and U is the velocity of the fluid. Within the fluid domain, it is assumed that the inner wall surface of the vessel-like flow channel is an immovable boundary. A mean blood pressure of 11 mmHg (1500 Pa) is taken in the venous flat position, and the pressure is fixed at the outlet [22]. Perfusion pressures of 12 mmHg, 13 mmHg, 14 mmHg and 15 mmHg were set at the inlet for the simulation. The hydrodynamic model was solved in a steady state, and the flow difference between the inlet and outlet of the flow channel model was monitored to ensure convergence of the simulation.

**Figure 4:**
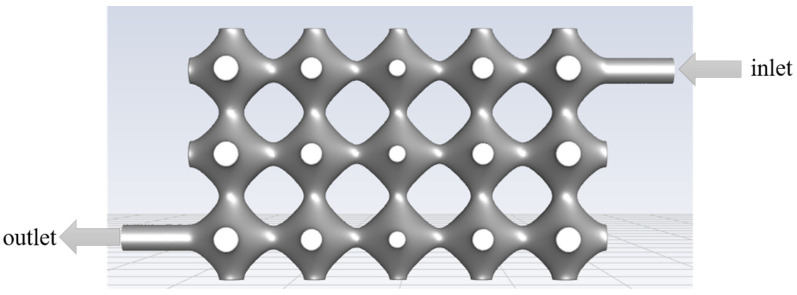
Fluid model entrances and exits.

The model was simulated using Fluent 2020 R2 (ANSYS, Canonsburg, PA, USA). The fluid inlet and outlet settings are shown in Figure 4, and the specific simulation parameters are set in Table 1.

## 3. Results

In Section 2 of the article, a finite element analysis of the tissue engineering in vitro perfusion process is performed for the simulation model developed. The simulation analysis is used to discuss the hydrodynamic properties of the flow channels during perfusion when the perfusion pressure is varied. The effect of perfusion pressure on blood flow rate and vascular flow channel wall pressure is analysed. Through simulation, we can optimise the tissue engineering perfusion parameters, as well as the 3D bioprintability of the model.

### 3.1. Effect of Different Filling Pressures on Pipe Wall Pressure and Fluid Velocity

**Figure 5:**
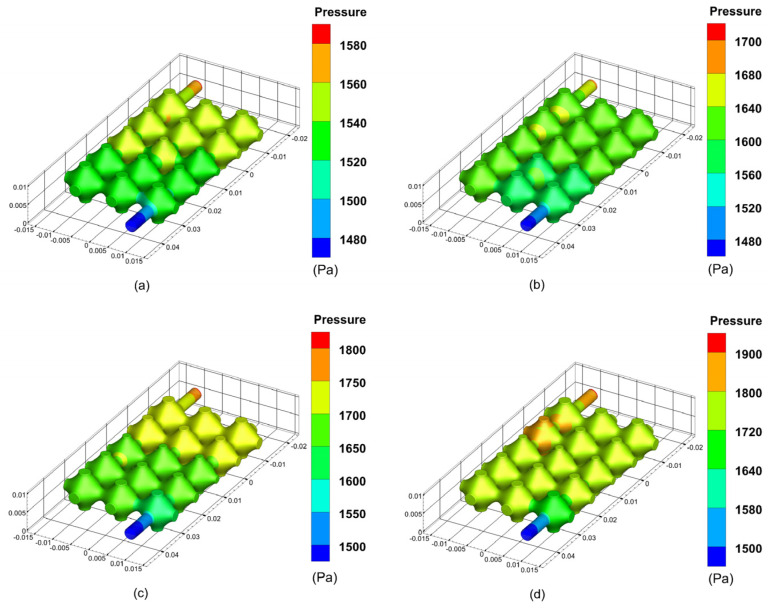
Results of wall pressure simulations of the class of vascular flow channels at different perfusion pressures: (**a**) 12 mmHg; (**b**) 13 mmHg; (**c**) 14 mmHg; (**d**) 15 mmHg.

During the perfusion of the vascular-like flow channel, hydrodynamic boundary loads exert pressure on the inner wall of the vessel. The results show that the average pressure in the vascular-like flow channel increases significantly with increasing perfusion pressure. Additionally, the vessel wall pressure is highest at the inlet and gradually decreases along the outlet direction, creating a pressure gradient and generating fluid flow dynamics. The simulation results of the pressure inside the flow channel of the class of vessels at different perfusion pressures are shown in Figure 5.

**Figure 6:**
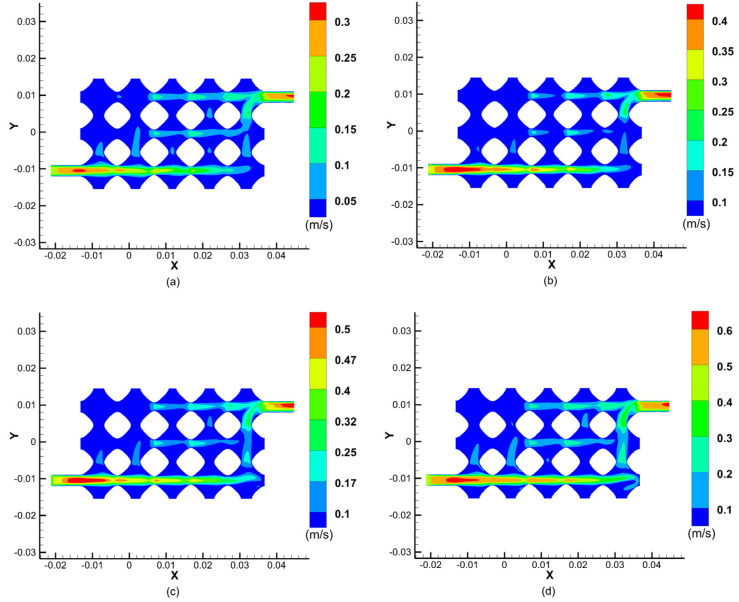
Simulation results of the fluid velocity at different perfusion pressures: (**a**) 12 mmHg; (**b**) 13 mmHg; (**c**) 14 mmHg; (**d**) 15 mmHg.

Figure 6 shows the results of a cross-sectional simulation of fluid velocities within the vascular-like flow channel at different perfusion pressures. The results show that the fluid velocity changes at different perfusion pressures, reaching a maximum at the centre of the inlet and outlet cross-sections and decreasing to a velocity near the inner wall of the vessel-like flow channel at 0. The blood velocity increases with increasing perfusion pressure, and as the perfusion pressure at the inlet increases from 12 mmHg to 13 mmHg, 14 mmHg, and 15 mmHg, the maximum values of the velocity at the centre of the inlet and outlet cross-sections increased from 0.3 m/s to 0.4 m/s, 0.5 m/s, and 0.6 m/s, respectively.

As can be seen from the graph of the fluid velocity cross-sectional simulation results, the fluid velocity in the upper left part of the model is almost zero, which indicates that the fluid flow through this part of the flow channel is relatively low, resulting in insufficient fluid flow rate to provide the corresponding material delivery rate to provide nutrients to the cells and carry away metabolites. This simulation provides a guide for the later improvement of the vascular-like flow channel model and subsequent successful perfusion.

### 3.2. Model Optimisation

**Figure 7:**
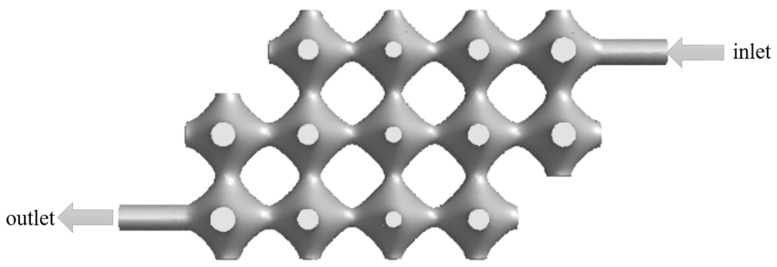
Optimised vascular-like flow path model.

To ensure proper tissue cell growth, the fluid must pass through each part of the model in order to provide nutrients and carry away metabolites. The model was optimised by removing the porous structure parts in the top left and bottom right corners of the model, and its optimised model is shown in Figure 7.

**Figure 8:**
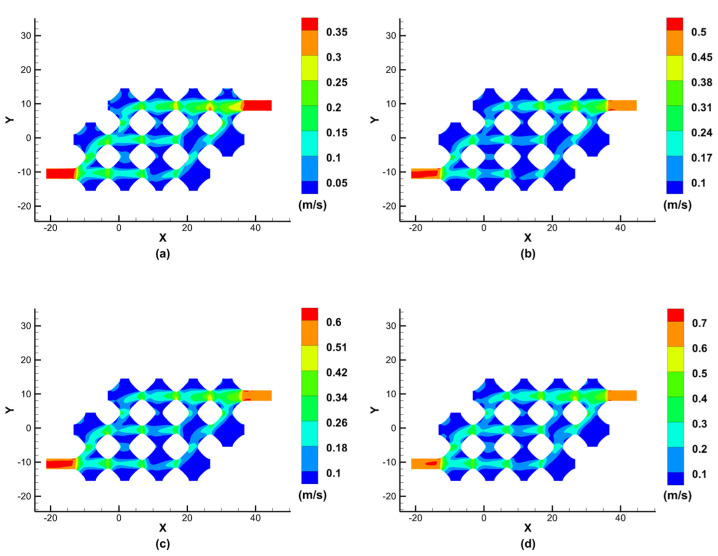
Simulation results of the fluid velocity at different perfusion pressures after optimisation: (**a**) 12 mmHg; (**b**) 13 mmHg; (**c**) 14 mmHg; (**d**) 15 mmHg.

For the optimised model, finite element simulations were carried out with the same parameters. The effect of perfusion pressure on blood flow velocity and vascular flow channel wall pressure was analysed. Figure 8 shows the results of the cross-sectional simulation of the fluid velocity in the class of vascular flow channels at different perfusion pressures after model optimisation.

As can be seen from the simulation results of the optimised model fluid velocity cross-section, when the inlet perfusion pressure is increased from 12 mmHg to 13 mmHg, 14 mmHg, and15 mmHg, the maximum velocity at the centre of the inlet and outlet cross-section increases from 0.35 m/s to 0.5 m/s, 0.6 m/s and 0.7 m/s, respectively. Although the removal of two parts makes the porous structure of the flow channel less porous, the remaining porous structures of the vascular flow channel all have fluid passing through them, which allows nutrients to diffuse and carry away metabolites, ensuring normal tissue cell growth. A reference value for the normal flow rate in the portal vein is 26 ± 14 cm/s, which means that the simulation results meet the requirements at an inlet perfusion pressure of 12 mmHg.

**Figure 9:**
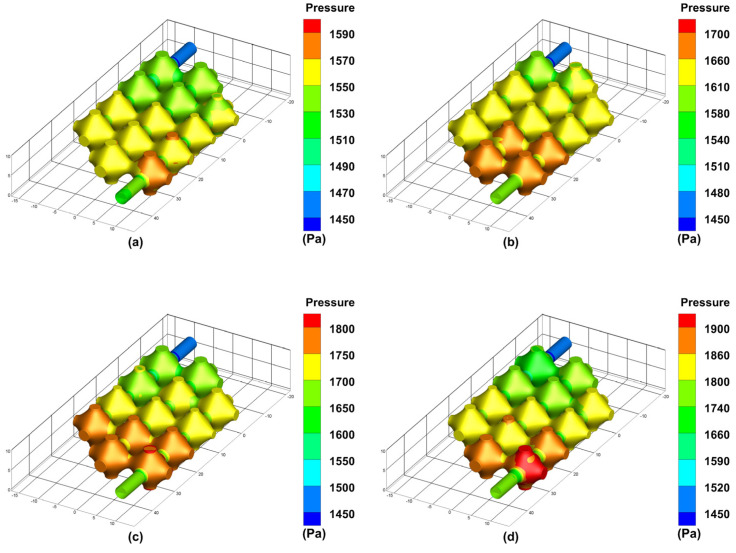
Simulated wall pressure results of the vascular-like flow channels at different perfusion pressures after optimisation: (**a**) 12 mm Hg; (**b**) 13 mm Hg; (**c**) 14 mm Hg; (**d**) 15 mm Hg.

The simulation results of the optimised flow channel model for the pressure exerted on the inner wall of the vascular-like flow channel at different perfusion pressures are shown in Figure 9. As can be seen from the simulation results, the average pressure of the porous structure is maximum near the inlet and minimum near the outlet. As the inlet perfusion pressure increases, the optimised pressure maxima in the vascular-like flow channel increase from 1590 Pa to 1700 Pa, 1800 Pa and 1900 Pa, with a significant increase in the pressure on the vascular-like flow channel walls. The venous pressure of the human body is very low, approximately 0–2660 Pa (0–19.9 mmHg) [23]. When the inlet perfusion pressure is 12 mmHg which satisfies the venous flow, the maximum inlet pressure is 1590 Pa, approximately 11.92 mmHg, which satisfies the human physiological parameters.

In our study, we focus on discussing and analysing the effect of perfusion pressure on blood flow rate and vascular flow channel wall pressure, and the results of our simulations guide optimising perfusion parameters. Nutrient fluid needs to flow through every part of the vascular flow channel of the porous structure of TPMS to ensure normal cell metabolism. However, as shown in Figure 6, the flow channel structure has the problem of no culture fluid flowing through some of the edge corner cells, which can lead to necrosis of the cellular tissue in this part without nutrient delivery. The improved flow channel model is shown in Figure 7, and the simulation results are shown in Figure 8. The improved flow channel model has nutrient flow through each part of the flow channel, improving the previous problem and ensuring normal metabolic activity of the cells, and the velocity simulation graph shows that the velocity is similar to the normal flow rate of human blood.

## 4. Discussion

This study shows the effect of the mechanical boundary load of the fluid on the pressure exerted on the inner wall of the vessel during the simulated perfusion process. The average pressure on the inner wall of the vessel increases with the increase in the perfusion pressure and gradually decreases from the inlet to the outlet direction, thus forming a pressure gradient, which provides a reference for the subsequent fabrication of the tissue flow channel. It also shows that the designed porous three-dimensional flow channel structure can better carry the traversal flow of nutrient solution, and its maximum specific surface area characteristic helps the tissue to carry out the sufficient nutrient exchange.

When artificial tissues are perfused in vitro, reasonable perfusion pressure is extremely important. Excessive perfusion pressure can lead to structural breakage of tissues, so based on suitable artificial tissue materials and maintaining a reasonable perfusion pressure, it is necessary to maintain the structural integrity of the internal flow channels of the tissues in order to achieve long-term tissue culture and viability. A simulation of tissue perfusion pressure in a vascularized flow channel was performed in the literature [19], which analyzed the effect of different properties of the tissue matrix material on the perfusion pressure leading to the vessel wall pressure. The material used for its tissue 3D printing was hydrogel, and the results showed that the hydrogel concentration had little effect on the flow rate and vessel wall pressure, while the cross-link density had a relatively small effect on the flow rate and vessel wall pressure, which was basically consistent with the concentration influencing factor. However, its tissue internal flow channel structure is too simple, which is more difficult to achieve effective simulation of biological tissues. The internal flow channel model tissue based on the TPMS structure proposed in this study can better simulate the characteristics of interflux structure inside the tissue and is more likely to realize the simulation of thick tissue structure in perfusion culture.

At the same time, we found that when artificial tissues are cultured by perfusion in vitro, the nutrient solution should flow through every part of the vascular flow channel as much as possible to maintain tissue cell nutrient and waste transport and ensure normal tissue cell metabolism for long-term culture and survival of artificial tissues. The literature [24] used a method of 3D bioprinting technology to construct a functional in vitro vascular channel with the perfused open lumen and the viability of tissues in a static cultured vascular channel, and the related findings can be used as a unique experimental tool to study the underlying mechanisms of vascular remodelling in the extracellular matrix and maturation processes under 3D flow conditions. However, the vascular structure of this study is only a cylindrical flow channel mechanism, which does not provide an effective simulation of the network structure of flow channels inside real tissues.

The ideal vascular flow channel structure requires nutrient flow through all regions of the tissue to facilitate nutrient delivery and waste excretion and also to facilitate cell attachment, such as endothelium. The TPMS structure-based internal flow channel organization model proposed in this study can simulate the internal network flow channel structure of thick tissues approximately and can realize the traversal network distribution to each region inside the tissues. Therefore, in the perfusion simulation study of this study, we investigated the influencing factors of flow rate, flow pattern and pressure based on the TPMS bionic vascular flow channel model to simulate the traversal delivery of the nutrient solution to each region of the tissues.

In this simulation study, we consider the cell perfusion culture fluid as a Newtonian fluid, i.e., a viscous fluid without solid particles, etc., in order to simplify the set of control equations. Moreover, this study only considered the effect of perfusion pressure on blood flow rate and vessel wall pressure and did not consider the effect of perfusion pressure on structural mechanics, such as the deformation of the vascular flow channel, which is a limitation of this paper.

## 5. Conclusions

In this study, a porous structured vascular flow channel model based on a minimalist surface is proposed. To ensure that the relevant parameters used in the in vitro perfusion culture are optimal, finite element simulations are performed to analyse the perfusion process of the porous vascular flow channel structured tissue model. The main objective of the simulation is to analyse the effect of perfusion pressure on blood flow rate and vascular-like flow channel wall pressure when the perfusion pressure varies. The simulation results of the initially designed model show that the blood flow velocity increases with increasing perfusion pressure when the perfusion pressure at the inlet is from, and the maximum value of the velocity at the centre of the inlet and outlet cross-sections increases gradually. The results show that the fluid velocity reaches the maximum in the centre of the inlet and outlet sections of the flow channel, the average pressure of the porous structure near the inlet is the highest, and the average pressure of the porous structure near the outlet is the lowest, and the overall pressure distribution shows a gradient variation, but there is a problem that no culture fluid flows through the porous unit, which located at the corners of some edges.

Through the analysis of the finite element simulation results, the vascular flow channel model is further optimised and improved. The simulation results of the optimised model showed that the maximum value of the velocity in the centre of the inlet and outlet sections gradually increased when the infusion pressure of the inlet increased, and the maximum value of the flow velocity in the centre of the optimised model was larger than that of the initial design model. Additionally, some areas in the initial design model almost fluid flow through, forming a dead zone for nutrient transport. The improved porous structure flow channel model has fluid flowing through all areas so that nutrients can fully diffuse the entire tissue model and take away metabolites, ensuring the normal growth of cells in human-like tissues.

The subsequent work in the article proposes to use simulation software to set fluid parameters for specific shear stress and flow patterns and feed the fluid into the designed TPMS flow channel model with certain flow values, where the fluid in the model can act on the cells on the tissue (e.g., endothelial cells) by the flow. Cell regulation and tissue development are achieved by selecting different flow patterns, such as laminar and pulsatile flow, interstitial flow, etc.

Compared with other structures, TPMS porous structure has gradient pore size variation, so it has stronger permeability and, therefore, has advantages in diffusion delivery of nutrients such as capillaries and small pore size restriction, which facilitates the diffusion of nutrients and ultimately promotes the development of tissue engineering in vitro culture.

## Figures and Tables

**Table 1:** Simulation parameter settings.

Volume Fill Cells Type	Poly
Viscous Model	Laminar
Blood Density	$1060\;\mathrm{kg}/m^{3}$
Hemodynamic Viscosity	$0.005\;\mathrm{Ns}/m^{2}$
Boundary Conditions	Stationary Wall, No slipping
Pressure-inlet	12, 13, 14, and 15 mmHg
Pressure-outlet	11 mmHg
Solution Methods	PISO
Solution Initialization	Hybrid Initialization

## Data Availability

Not applicable.
